# Evaluation of the Long-Lasting Flavour Perception after the Consumption of Wines Treated with Different Types of Oenological Additives Considering Individual 6-n-Propylthiouracil Taster Status

**DOI:** 10.3390/foods12152835

**Published:** 2023-07-26

**Authors:** Rafael I. Velázquez-Martínez, Celia Criado, Carolina Muñoz-González, Julia Crespo, María Ángeles Pozo-Bayón

**Affiliations:** 1 Instituto de Investigacion en Ciencias de la Alimentacion, CSIC-UAM, Nicolas Cabrera 9, 28049 Madrid, Spain; rafael.v.m@csic.es (R.I.V.-M.); celia.criado@csic.es (C.C.); c.munoz@csic.es (C.M.-G.); 2 Departamento de Investigación Agroambiental, Instituto Madrileño de Investigación y Desarrollo Rural, Agrario y Alimentario (IMIDRA), El Encín, A-2 km 38.2, 28805 Alcalá de Henares, Spain; julia.crespo.garcia@madrid.org

**Keywords:** wine, oenotannins, mannoproteins, flavour persistence, PROP taste phenotype, time–intensity sensory analysis

## Abstract

Due to the limited scientific knowledge on the impact of commercial oenological additives on flavour perception, the aim of this work was to evaluate the effect of different types of oenological additives on the long-lasting flavour perception (flavour persistence) during wine tasting, also considering the effect of the individual PROP (6-n-propylthiouracil) taster status (PTS). To do so, white and red wines with two oenotannins (ellagitannin and gallotannin) and a commercial yeast mannoprotein were prepared. A control wine of each type was also made without additives. All the wines were spiked with a mixture of aromatic compounds responsible for the “fruity” and “woody” notes. Retronasal aroma and astringency were evaluated at the same time using time–intensity (TI) methodology and a trained panel (n = 40), including PROP non-tasters (NTs) and tasters (Ts). The results showed a significant effect of PTS on the long-lasting perception of astringency, being Ts who showed higher values than NTs for most TI parameters. However, PTS did not affect aroma persistence. In addition, the three oenological additives had an effect on astringency and retronasal aroma perception. They significantly increased the long-lasting perception of astringency compared to the control, while gallotannin also increased the persistence of the woody aroma.

## 1. Introduction

The use of natural additives to improve the winemaking process is becoming a current practice in the oenological industry, leading to a large variety of commercial additives with different claims related to many technological and sensory properties in wines. Nonetheless, the number of scientific studies supporting the potential benefits of these compounds and their optimal usage during winemaking is still quite scarce. In general, most of these additives come from different sources, being oenotannins and yeast derivatives among the most commonly used. 

Oenotannins can come from grapes or other different botanical sources. The International Organization of Vine and Wine (OIV) defines their use for the stabilisation and fining of musts and wines, as well as to increase the antioxidant and antioxidasic capacity of grape juice and to promote colour stability (resolutions OIV-OENO-612–2019 and OIV-OENO-613–2019). Nonetheless, besides these effects, they can also affect some wine sensory properties such as aroma, astringency, and bitterness [1,2,3,4,5,6]. This mainly depends on their chemical structure and concentration [7,8]. Depending on their chemical characteristics, oenotannins can be generally divided into condensed tannins (or proanthocyanidins) and hydrolysable tannins. Proanthocyanidins can be polymers of flavan-3-ols and flavan-3,4-diols. In grapes, they are flavan-3-ols-based polymers, namely procyanidins, which are composed of (+)-catechin and (−)-epicatechin with different extents of galloylation [9]. Additionally, there are condensed tannins with flavan-3,4-diols subunits extracted from exotic woods (quebracho, acacia, etc.) for oenological use [7,8]. Regarding hydrolysable tannins, they can be classified as gallotannins and ellagitannins. Gallotannins are present in plant gallnuts, and they are composed of gallic acid and D-glucose, with different extents of substitution with galloyl moiety. Meanwhile, ellagitannins are formed by D-glucose and ellagic, gallic, or hexahydroxydiphenic acids, and they are usually extracted from chestnut and oak [10].

Besides oenological tannins, yeast derivative products constitute a second type of oenological additives widely used during winemaking. They include different products such as inactivated yeast, inactivated yeast with glutathione, autolysate, yeast protein extract, and yeast wall mannoproteins [11,12]. Traditionally, they have been used to provide assimilable nitrogen or to stimulate yeast and lactic bacteria growth and prevent stuck fermentations [11,12]. Currently, mannoproteins are also being used for increasing wine colloidal stability, which is an application supported by the OIV (resolution OIV-OENO 417–2011). Nonetheless, the use of yeast derivatives in wines has also been shown to have additional effects on wine sensory characteristics. For instance, they preserve the intensity of the colour of wines, eliminate the excess of tannins related to wine astringency, and also have an impact on wine aroma [13,14,15,16,17,18,19].

Interestingly, tannins and mannoproteins are also polymers with the ability to bind the oral surfaces (mucoadhesion properties). For instance, Ginsburg and collaborators [20] showed that tannins can be retained in the oral cavity for long periods despite a constant salivary flow. More recently, it has been proven the existence of intermolecular interactions between tannic acid and mucin [21]. These interactions affect the retention and release of aroma compounds in the oral cavity [22,23,24,25].

In the case of mannoproteins, recent works using salivary proteins, protein-rich proline proteins (PRPs), and a cell-based model of the oral epithelium system also show that mannoproteins can interact with some salivary proteins and tannins, with an impact on astringency modulation [26,27,28]. Besides this, well known is the existence of interactions between aroma compounds and mannoproteins, which depend on the hydrophobicity of the aroma compounds and on the composition of the mannoprotein (glucidic/protein ratio) affecting aroma release, as shown when using headspace analysis [15,16,29].

Given the ability of tannins and mannoproteins to be retained on the oral surface together with the capacity of both types of polymers for binding aroma molecules, the hypothesis arises that they can have a preponderant role in flavour persistence, which is the long-lasting perception of flavour stimuli produced immediately after wine swallowing [30]. This phenomenon is a key factor in the sensory experience of consumers and therefore very much related to wine preference and liking. 

In this sense, it is also important to notice that wine flavour perception, including flavour persistence, might be greatly variable depending on many types of genetic, biological, physiological, and psychological factors [31]. Among them, taste phenotype, or PROP taster status (PTS), measured as the sensibility to taste the bitter compound 6-n-propylthiouracil (PROP), has been one of the most studied [32]. It has been described that PROP taste individuals also have a higher acuity to perceive other basic tastes, mouthfeel (astringent), and olfactory stimuli [33]. Although still under study, the higher sensory ability of PROP taste individuals has been related to many different factors, such as differences in TAS2R38 gene polymorphisms, differences in fungiform papillae density, salivary protein composition, or age and gender, among others [34]. Interestingly, in previous studies on wine, the association between PTS and a higher sensory ability to perceive taste, mouthfeel, and olfactory stimuli has been rather controversial, with some studies showing a positive correlation [35] while others do not [36,37,38].

Considering the lack of specific studies focused on the effect of oenological additives on wine flavour persistence, the objective of this work was to test if three common commercial oenological additives, namely, hydrolysable tannins (gallotannin and ellagitannin) and yeast mannoproteins, might affect the long-lasting flavour perception (astringency and retronasal aroma) of wines (red and white). For this purpose, a dynamic sensory technique (time–intensity) was used, which is based on scoring the evolution of the intensity of the flavour stimulus (fruity and woody aroma and astringency) from the moment it appears (immediately after the wine is tasted) until it is no longer perceived. Additionally, the effect of PTS (ability to perceive the bitter compound 6-n-propylthiouracil) was also tested by using a trained panel formed of tasters (n = 20) and non-tasters (n = 20).

## 2. Materials and Methods

### 2.1. Wine Samples

A red wine and a white wine from Tempranillo and Malvar grape varieties were industrially produced at the IMIDRA experimental winery (Alcalá de Henares, Madrid, Spain). These wines were considered the control wines (red, CRW; white, CWW). Their chemical composition is shown in Table S1. From each control wine, three more wines were formulated by adding three types of commercial oenological additives (Table 1). One of them was a mannoprotein from yeast wall and the other two were oenological hydrolysable tannins, specifically a gallotannin and an ellagitannin. Their chemical compositions are shown in Table S2. All of them were provided by Laffort Ibérica S.A. They were added before bottling at the concentration recommended by the manufacturer for wine applications. The wine types and the final concentration of each additive in the wine are shown in Table 1. 

### 2.2. Wine Aromatisation

To reinforce the aroma profile of the wines in two aroma descriptors of interest (fruity and woody), before the sensory test, all the wines were independently aromatised with two aroma mixtures, responsible for these aroma nuances using food-grade odorant compounds (Table 2) from Merck (Darmstadt, Germany). For the preparation of the aroma solutions, individual solutions of each aroma compound responsible for the fruity and woody aroma notes were weighted and diluted in food-grade ethanol. From this, a working stock solution containing all the aroma compounds of each aroma mixture was prepared. Two hundred microliters of this solution was added to each wine (15 mL contained in a wine glass) before each test to have the final concentrations shown in Table 2. These concentrations were chosen in previous lab trials, considering that they should be easily distinguished by the panel but not unpleasant, which might negatively affect the completion of the test. Likewise, the aromatic concentrations were taken as a reference from previously published works [38]. Each aroma mixture was independently poured and evaluated in all four wine types (reds and whites). Aromatisation was performed 5–10 min before the beginning of the sensory evaluation. During this time, the wine glasses were covered with plastic Petri dishes to prevent volatile loss.

Therefore, a total of sixteen wines were produced: (a) eight red wines: control wine without and with the three additives aromatised with fruity and woody aroma mixtures, and (b) eight white wines produced under the same conditions (Table 1). 

### 2.3. Individual Panel 

Forty individuals from two different PROP taste phenotypes (PROP tasters and non-tasters) (see Section 2.4) were recruited for this study. Additionally, the inclusion criteria for participation were healthy, non-pregnant, and adult volunteers (over 18 years old). In addition, all volunteers completed a food allergy screening document, which included allergy/intolerance to wine or any of its components. Each volunteer attended one-hour sessions eight times. All the participants were informed of the nature of this study and gave written consent to participate. This work was approved by the Bioethics Committee of the Spanish National Research Council (CSIC, 008/2021). This study was conducted in April and May 2022 at the CIAL (Madrid, Spain). 

### 2.4. Taste PROP Phenotype

Individual taste PROP phenotype was tested using commercial strips impregnated with 6-n-propylthiouracil (3 µg/strip) from Sensonic International (Haddon Heights, New Jersey). Triangular tests were conducted with two blank samples (no impregnated strips) (Sensonic International) and one impregnated sample to test whether consumers were able to recognise the PROP sample. If the volunteers did not perform the triangular test correctly, they were considered to be in the non-taster group. Individuals who positively recognised the PROP sample were retested and they evaluated the perceived intensity, which was assessed using the Generalised Labelled Magnitude Scale (gLMS) scale (0–100; from “no sensation” = 0, to “strongest imaginable sensation of any kind” = 100) [39]. For each sample, they were instructed to swipe the paper strip across the tongue, remove the strip, press the tongue against the roof of the mouth, and swallow. Between samples, they were asked to drink water. Individuals were classified into two groups: non-tasters (NTs; n = 20) and tasters (Ts; n = 20).

### 2.5. Dynamic Sensory Analyses 

#### 2.5.1. Training 

The absence of anosmia was previously confirmed by means of a triangular test with flavoured hydroalcoholic solutions. Individuals received specific training for the recognition of fruity and woody aromas as well as astringency using red and white wines. In the following sessions, training sessions for the recognition of the intensity of the different sensory stimuli perceived with the different types of wine were also carried out. Additionally, during these sessions, individuals were instructed in the use of the intensity scale (15 cm unstructured scale delimited at the ends), and in the TI methodology using tablets for sensory data collection. 

Samples (15 mL) were served in standard tasting glasses (20 mL) covered with plastic Petri dishes to avoid loss of volatiles. For this purpose, wine glasses were labelled with random 3-digit codes and presented simultaneously in a randomised order using a Balanced Complete Block design [40]. Mineral water (Aliada, Madrid, Spain) and breadsticks (El Corte Inglés, Cádiz, Spain) were offered to cleanse the palate. 

Sensory evaluation sessions were conducted using Compusense^®^ Cloud software (Compusense Inc., Guelph, ON, Canada) via tablets, where each consumer had a user profile and could access each of the tests.

#### 2.5.2. Sensory Evaluation

For the evaluation of the sensory stimuli (retronasal aroma and astringency), panellists gently rinsed their mouths with the wine (15 mL) for 30 s, then spat it out. During rinsing, special care was taken to keep the lips closed, not to swallow and not to open the velum–tongue border prior to expectoration. Then, they were instructed to swallow the remaining saliva in their mouth and to start the TI evaluation. For this, panellists moved the cursor along the unstructured scale (15 cm) to evaluate the astringency and aroma intensity perceived (of one single aroma attribute) that lasted for two minutes. The evaluation of the intensity of both stimuli in the same trial avoided the halo-dumping effect [41,42]. Data were recorded at a frequency of 1s.

#### 2.5.3. TI Data Analyses

For each sensory stimulus, TI curves were obtained by averaging the data at each point of time across the two groups of subjects (Ts and NTs). The raw data were obtained by Compusense software. Moreover, four typical time–intensity parameters were extracted from the TI curves using the XLSTAT Sensory software: time to reach the maximum intensity (Tmax), maximum intensity (Imax), duration time of the perceived stimuli (Tend), and area under the curve (AUC).

### 2.6. Statistical Analyses 

Analysis of variance ANOVA (one-way or two-way) and mean comparison tests (Tukey) were applied to check the effect of PROP taste phenotype and the effects of the oenological additives on the different parameters (Imax, Tmax, Tend, AUC) extracted from the astringency and aroma TI curves. A significance level of *p* < 0.05 was always used. Statistical analyses were carried out using XLSTAT (Version 2019.01).

## 3. Results

### 3.1. Effect of PROP Taste Phenotype on Flavour Perception in Red and White Wines

PROP taster status (PTS) has been associated with a higher individual ability to perceive sensory stimuli from wines [35]. However, there were no previous studies that tested this using dynamic sensory methods that better represent the flavour perception experienced during wine consumption. Therefore, the effect of PROP phenotype on wine astringency and retronasal aroma perception over time was first checked.

#### 3.1.1. Effect of PROP Taster Status in Wine Astringency Perception over Time

**Figure 1:**
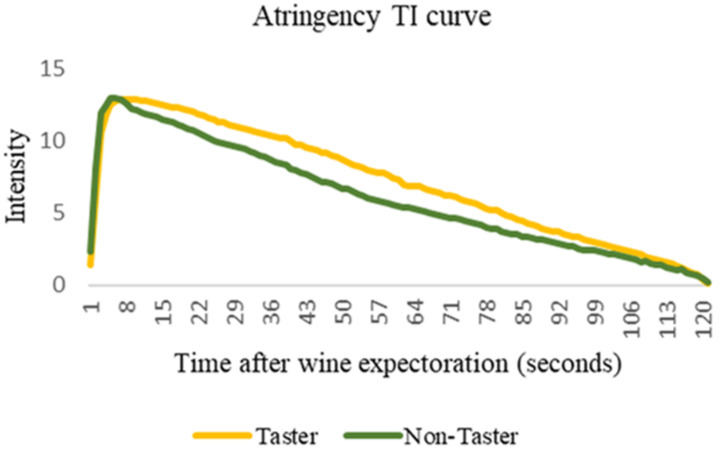
Example of the time–intensity average profile (n = 20) of astringency obtained during the tasting of the fruity flavoured red wine considering both PROP taste phenotypes (taster and non-taster).

As can be seen in Table 3, in the case of astringency, tasters (Ts) showed significantly higher values of Text and AUC in the case of red wines with a woody aroma. For instance, T phenotypes showed values of Text and AUC that were 9.7% and 18% higher compared to NT. The same results were found in the case of the red wine with a fruity aroma. In this case, Ts also exhibited higher values for most TI parameters. For instance, Tmax, Text, and AUC values were 26.9%, 9.6%, and 21% higher, respectively, compared to the non-taster (NT) phenotype. Figure 1 shows the TI curves (average values from 20 volunteers) for the astringency perception obtained for this wine considering both PROP taste phenotypes. 

In the case of white wines, there were also significant differences in the perception of astringency depending on PROP taster status, but only in white wines aromatised with fruity aroma notes. As occurred in red wines, in white wines, Ts showed higher T max values (40% more) than NTs. On the contrary, Ts showed 16.8% lower values of Imax compared to NTs. There were no differences between PROP phenotypes in the other TI parameters, showing a lower impact of the perception on the astringency in the case of white wines.

#### 3.1.2. Effect of PROP Taster Status on Wine Retronasal Aroma Perception over Time

As shown in Table 4, in the case of both retronasal aroma attributes (woody and fruity aroma), there were no significant differences based on PTS in any of the parameters extracted from the TI curves in neither red nor white wines. 

### 3.2. Effect of the Oenological Additives on Flavour Perception in Red and White Wines

In a further step of the work, the effect of the oenological additives in the perception of the astringency and retronasal aroma (fruity and woody) over time was also checked. 

#### 3.2.1. Effect of Oenological Additives in Wine Astringency 

Results corresponding to the differences in TI parameters considering the control wine and the wines supplemented with oenological additives are shown in Table 5. Since, as shown before, the evaluation of astringency was affected by the individual PROP taste phenotype, Table 5 shows these results considering both taste phenotypes. 

As can be seen in Table 5, the effect of the oenological additives on astringency was significant (*p* < 0.05) in the case of red and white fruity wines, but not in the case of wines aromatised with a woody aroma. In the case of red fruity wines, for both PROP taste phenotypes (T and NT), wines with oenological additives exhibited lower Tmax values compared to the control. The lower Tmax was more pronounced for Ts (60% lower compared to the control) than for NTs (34.6% lower compared to the control). A lower Tmax value means that the maximum intensity of perceived astringency will be quicker in wines with additives, and even faster for individuals of the T phenotype.

In the case of fruity white wines, the effect of oenological additives was only significant (*p* < 0.05) for Ts. Interestingly, they also exhibited higher Imax values of astringency for the three wines with additives compared to the control wine. Of the three additives, mannoproteins provoked the largest effect, increasing Imax by 27.4% compared to the control wine. On the contrary, the impact of the additives was not significant for the other TI parameters, nor in the case of wines (red or white) with a woody aroma.

#### 3.2.2. Effect of Oenological Additives on Retronasal Aroma 

To check the effect of the oenological additives on TI parameters obtained from the retronasal aroma evaluation, data from both PROP taste phenotypes were considered together, since, as previously shown, this factor did not affect TI retronasal aroma evaluation.

As shown in Table 6, only fruity red wines with additives exhibited significantly lower (above 30%) T max values compared to the control wine. Similar results were found in the case of fruity white wines, although in this case, the effect of the additive was slightly different. Although the wines with additives exhibited lower Tmax values compared to the control, a higher effect was noticed in the wine with mannoprotein, in which a reduction in Tmax of 48% was found. These results mean that the three additives reduced the time to reach the Imax, and therefore, their addition in wines produces a quicker fruity sensation, which is more pronounced in the case of mannoprotein in white wine. Interestingly, white wines with mannoprotein also exhibited higher Text and AUC values, although these results were not significant.

Additionally, the results in Table 6 also show that in white wines with a woody aroma, the addition of gallotannin increased the AUC (18.6% more than the control), which is related to the total aroma perceived, while on the contrary, the addition of mannoprotein reduced this parameter (18.8% less than the control). 

## 4. Discussion

The main objective of this work was to evaluate the effect of three widely used commercial oenological additives of different natures, based on hydrolysable tannins (gallotannin and ellagitannin) or yeast mannoproteins, on the long-lasting flavour (astringency and retronasal aroma) perception (also called wine flavour persistence) of red and white wines. Besides this main objective, the study also focused on testing if individual PROP taste phenotype (or PROP taster status, PTS) might have an effect on flavour persistence. The results of this study show that PTS has a significant impact on astringency perception, mainly in red wines. In this sense, the analysis of the parameters extracted from the TI curves (Table 3) confirmed that taster individuals (individuals able to perceive the bitter compound 3-propylthiouracil, PROP) showed higher values of most TI parameters, such as Tmax, Text, and AUC. Tmax is related to the time necessary for reaching the maximum intensity of astringency (Tmax), while Text is related to the time required until the extinction of this sensation (Tex). Therefore, Text is directly related to the long-lasting perception of astringency. Furthermore, higher AUC values are related to a higher overall astringency perception. Therefore, the results from this work show that the persistence of astringency was higher in individuals classified as PROP tasters. For instance, in red wines, AUC values were significantly higher (about 21%) in PROP taster individuals compared to non-tasters (Figure 1). These results were, as indicated, more relevant in red wines than in white wines. In white wine, PROP taster individuals only exhibited the highest Tmax values of astringency in the fruity aromatised wine, but these differences were not noticed in the woody white wine (Table 3). Even PROP taster individuals showed lower Imax values when tasting this wine type compared to non-taster individuals. These differences could be linked to the higher astringency of red wines, which are generally richer in natural astringent phenolic compounds compared to white wines. Since astringency is lower in white wines, this sensation could have been more difficult to distinguish and to be rated by the panel (independently of the taste phenotype) in white wines compared to red wines.

Interestingly, previous works with wines have related the individual ability to detect the bitterness of PROP with a higher ability to detect other basic tastes (sweet, salty, acid) and trigeminal sensations such as astringency [35]. Nonetheless, pioneer works on the topic [43] did not show an association between PROP phenotypes and the ability to perceive astringency. This question has been somehow quite controversial in the scientific literature. While Pickering and co-workers did confirm this association [35], in more recent works, the same authors and others did not show a relationship between PROP phenotype and wine astringency perception [36,37,38]. Some of the reasons suggested to explain this lack of agreement were that astringency is a sensation that evolves over time, and therefore, differences among PROP taste phenotypes could be masked when astringency is only rated considering a single time point [36]. The results from the present work seem to confirm this hypothesis, and to the best of our knowledge, this is the first time that the difference in the ability to perceive wine astringency between PROP taster and non-taster individuals has been confirmed using a dynamic sensory approach, evaluating the development of this mouthful sensation from its onset until it is no longer perceived.

Additionally, some previous works also suggested that the higher sensory ability of PROP taster individuals could also include a higher retro-olfactive performance [33]. Results from the present study using two types of aroma mixtures representing congruent aroma notes of red (woody aroma) and white (fruity) wines do not confirm this hypothesis. Neither in white wines with woody or fruity aromas nor in red wines eliciting the above-mentioned aroma nuances were there significant differences in any of the TI parameters when considering PROP taster and non-taster individuals. Although the genetic and physiological mechanisms behind differences in sensitivity among taste PROP phenotypes are not sufficiently understood [34], some positive correlations such as a higher number of taste papillae and greater trigeminal innervation are often positively linked to PROP taster individuals [34,35]. These factors, however, seem to be more related to taste and trigeminal sensations than to olfactive inputs, which could explain the lack of an effect of PROP taste phenotype on retronasal aroma perception. On the contrary, the sensation of astringency involves the activation of mechanoreceptors in the oral cavity, which themselves are innervated by trigeminal fibres, and therefore, the effect of taste PROP phenotype could be much more relevant to taste or mouthfeel sensations than when considering olfactive stimulus [35].

A second objective of the work was to check if the use of typical oenological additives in wines such as oenotannins and mannoproteins might have an impact on the long-lasting flavour perception. Although these types of additives have long been used and represent a common technological practice currently available for winemakers, the scientific studies devoted to understanding their sensory impact are relatively scarce, and to the best of our knowledge, there is no previous study devoted to knowing their impact on flavour persistence. 

The results from the present work show an impact of these additives on wine astringency in red and white wines, but only in those wines aromatised with a fruity aroma mixture and not in the case of the wines aromatised with a woody aroma (Table 5). This could be related to the existence of aroma–astringency interactions at a cognitive level, which can modify astringency perception [44,45]. For instance, in previous works [46], authors have shown that the fruity aroma extracted from a Chardonnay wine and added to a dearomatised red wine induced a lower astringency in the latter. However, more recent works [47] did not find an effect of different categories of odours on astringency perception, although they did not specifically evaluate the effect of a woody odour mixture. Another possible explanation is that the task of rating astringency in fruity wines could have been easier for the panel than in woody wines, considering that this odour is more associated with a mouthfeel sensation such as astringency. 

In the case of fruity red and white wines, the addition of additives induced small but significant changes in the astringency perception. In general, although the overall astringency perception, intensity, and duration were not modified by these additives, other TI parameters such as Tmax were significantly affected. Nonetheless, the effect was different in red and white wines. For instance, in red fruity wines, all the additives provoked significantly (*p* < 0.05) lower Tmax values compared to the control (Table 5). This means that the time necessary to perceive the maximum astringency is shorter when adding these additives. The drop in Tmax was more pronounced for individuals belonging to the T phenotype (60% lower compared to the control) than in the individuals from the NT phenotype (34.6% lower compared to the control), supporting the idea that the higher sensitivity of taster individuals allowed them to detect the changes induced by the additives to a larger extent compared to the NT group. Nonetheless, in the case of white fruity wines, the additives significantly increased the astringency Imax compared to the control, but this was only observed in the taster phenotype, adding scientific support to the hypothesis that taster phenotypes might be more sensitive to the changes in some sensory stimuli (e.g., astringency) induced by this type of winemaking practice. Additionally, in the case of white fruity wines, the addition of mannoproteins induced the highest Imax values (Table 5). It is interesting to note that although some previous works have shown that commercial mannoprotein-rich yeast extracts reduce wine astringency [48,49], other works did not find any effect, even when they were added at very high concentrations to the wines (6 g/L) [50]. Nonetheless, it is worth noting that these previous works evaluated this mouthfeel sensation through descriptive sensory analysis at a single point after wine tasting, but not the astringency persistence like in the present work, which might lead to very different results. Additionally, as shown before, there are many types of mannoproteins that can vary in their glucidic and protein content, and, therefore, in their chemical and sensory properties [51], which might induce different effects in wines. Even the wine composition can have an influence on the role of mannoproteins in astringency and other wine sensory properties [19]. Although mannoproteins induced the highest Imax values for astringency in white fruity wines, a slightly lower but significant effect on Imax was also observed after the addition of the polyphenol-based additives (quertannin and gallotannin) (Table 3), which could be related to the increase in the phenolic content of the wine, which, in turn, can affect wine astringency, as recently shown [24].

Regarding the effect of the oenological additives on aroma persistence, even though a significant effect was observed, this effect was different depending on the wine type (red or white) but also depending on the aroma considered (fruity or woody). In this sense, the addition of additives in red fruity wines decreased Tmax (above 30%) compared to the control wine (Table 6). This means that the fruity intensity will be perceived earlier or faster in wines with additives. On the contrary, this effect was not observed when the panel considered the same wine but aromatised with a woody aroma. This agrees with the results related to the lack of an impact of additives on astringency in wines (red or whites) aromatised with a woody flavour, supporting the hypothesis of the existence of woody aroma–astringency interactions, which might affect the performance of the panel in the evaluation of either astringency or retronasal woody aroma. Additionally, other explanations, such as physicochemical interactions between the additives and the volatile compounds included in the aroma mixtures, cannot be discarded. The different chemical compositions of the fruity and woody aroma mixture might have induced different types of interactions (aroma–polyphenol, aroma–mannoprotein) with the additives, affecting aroma release, and, therefore, aroma perception [15,16,29,52,53].

In the case of white fruity wines, the effect of the additive or retronasal aroma was similar to that found for the fruity red wine, that is, a reduction in Tmax compared to the control (Table 6). Nonetheless, in this wine, the effect induced by the mannoprotein was significantly higher (48% lower Tmax compared to the control wine) than the effect produced by the polyphenol-type additives. Additionally, white fruity wines with mannoproteins also exhibited the highest Text values from the four tested wines (control wines and wines with additives), although these results were not statically significant (Table 6). Interestingly, in a previous work, an increase in fruity, floral, and balsamic aromas was found in Sangiovese wines aged for six months in contact with three different commercial mannoprotein-rich yeast extracts at a concentration of 20 g/hL [19]. Considering the large diversity of these types of additives, the results found in this work are interesting since, besides the quicker fruity perception that mannoproteins are able to induce, they also could improve and extend the fruity perception in white wines over time, which is an interesting technological feature associated with this type of additive, which will be necessary to investigate in the future. 

On the other hand, besides the effect of the additives on the fruity aroma perception of white wines, they also had an effect on the woody aroma persistence. For instance, the addition of gallotannin in white woody wines significantly increased AUC compared to the control (Table 6), while the addition of mannoprotein produced the opposite effect, a decrease in AUC compared to the control. The addition of ellagitannin did not produce a significant effect on woody aroma persistence, which was very similar to that of the control wine. The addition of gallotannins could have incorporated some volatile compounds into the wine, contributing to an increased overall woody aroma. This effect has been recently shown when incorporating ellagitannin extracts into wines [24]. Additionally, the gallotannins could have an effect on wine astringency, which, as previously described, could induce taste–aroma interactions at a cognitive level and a higher perception of woody notes usually associated with astringent wines. This idea could be also valid to explain the reduction in woody aroma persistence (AUC) compared to the control induced by mannoproteins in the white woody wine (Table 6). It is known that yeast polysaccharides modify the aggregation between tannins and proteins, affecting astringency through two possible mechanisms: (i) competition between polysaccharides and salivary proteins towards tannins and (ii) polysaccharides forming a ternary complex, protein–polyphenol–polysaccharide, which enhances solubility in an aqueous medium [51]. As previously explained, the reduction in this mouthfeel sensation could be related to a lower perception of woody aroma, often associated with astringency. This seems a plausible explanation, since, as shown in Table 5, the lowest values of AUC (indicative of the total astringency perception) in white wines with a woody aroma were found when mannoproteins were added. 

Besides an effect at the cognitive level, other explanations, involving physicochemical interactions between the additives and the aroma compounds included in the aroma mixture, could also contribute to explaining the opposite effect of gallotannin and mannoproteins on the woody persistence in white wine. In this sense, it has been previously shown that the addition of some kinds of polyphenolic extracts, especially flavan-3-ols, increased oral aroma persistence, therefore prolonging the release of certain volatile compounds by the tertiary interactions among oral mucosa, polyphenols, and aroma compounds [28]. These associations can form aroma reservoirs at the surface of the oral mucosa, ready to be released by the respiratory air flows several minutes after swallowing the wine [25,54]. More recently, it has been proven the existence of intermolecular interactions between tannic acid and mucin, which indeed affect the retention and release of aroma, suggesting that these types of polyphenols have the ability to delay aroma release, prolonging aroma perception [20]. Additionally, Pittari and co-workers [24] also show that hydrolysable tannins (ellagitannin) in non-oxidised wines increased the in-nose release of wine volatiles. However, in this work, the authors did not evaluate aroma persistence by sensory analysis. 

The fact that the addition of ellagitannin did not induce a higher aroma persistence like gallotannin did shows the necessity of more studies comparing chemically different types of oenotannins in order to unravel the molecular action mechanisms which might help in the development of additives with better technological properties for improving aroma persistence.

Additionally, the existence of physicochemical interactions between mannoproteins and aroma compounds can also explain the reduction in aroma persistence that these yeast polysaccharides produced in white woody wines. In fact, previous studies showed hydrophobic interactions between mannoproteins and aroma compounds, mainly of hydrophobic nature, able to reduce aroma release [15,16]. Considering that woody aroma is composed of some relatively higher hydrophobic compounds (e.g., vainilline, whiskylactone) (Table 1), a large retention of these compounds on mannoproteins could be expected. Depending on the degree of interaction, oral aroma release could be slowed down compared to the control wine, translating into a lower overall aroma perception. Nonetheless, these results show the necessity of new in vivo analytical and sensory studies including these types of additives from different sources and with different chemical properties in order to confirm their role in aroma persistence and their usefulness to improve wine aroma persistence.

## 5. Conclusions

Results from this study using time–intensity methodology confirm the significant effect of individual PROP taste phenotype on the long-lasting astringency perception after wine consumption. PROP taster individuals (those able to perceive the bitter compound 3-propylthiouracil, PROP) showed higher values for most TI parameters (Tmax, Text, and AUC) compared to non-taster individuals. The effect of taste phenotype was more evident in red wines than in white wines. Nonetheless, PROP phenotype did not affect the long-lasting aroma perception of the tested aromas (fruity and woody) in either red or white wines. Additionally, the results showed that common oenological additives of different natures, such as hydrolysable tannins (gallotannin and ellagitannin) and yeast polysaccharides (mannoproteins) added at recommended concentrations to red and white wines have an impact on flavour persistence, affecting astringency and retronasal aroma perception. This effect depends on the wine type (red or white) and the type of aroma considered. For instance, they affect the astringency perception of red and white wines, but only in those wines with a fruity flavour. In red wines, they reduce the time to reach the maximum astringency intensity (Tmax), while in white wines, they induce a higher maximum intensity. In the case of aroma persistence, in red and white fruity wines, the addition of these additives allows for reaching the maximum intensity of fruitiness sooner. This was especially relevant in the case of mannoproteins in white fruity wines. On the contrary, none of the additives have a significant effect on the woody aroma in red wines, while in white wines, the addition of gallotannin significantly increased the global woody aroma perception (AUC) compared to the control. Overall, the wide diversity of chemical structures and properties of oenotannins and mannoproteins offers a great opportunity to use them as oenological tools to improve and/or modulate the aromatic persistence of wine. However, further in vivo analytical and sensory studies will be necessary to select the ones that provide the best results.

## Figures and Tables

**Table 1:** Wine types and concentration used of each oenological additive.

Oenological Additive	White Wines ^a^		Red Wines ^a^	
	Wine Type (f/w)	Concentration	Wine Type (f/w)	Concentration
No additive (control)	CWW	--	CRW	--
Gallotannin	GTWW	300 mg/L	GTRW	300 mg/L
Ellagitannin	ETWW	700 mg/L	ETRW	700 mg/L
Mannoprotein	MWW	1.5 mL/L	MRW	0.9 mL/L

^a^ All the wines (white and red) with and without oenological additives were aromatised with a fruity and a woody aroma mixture. Therefore, 8 types of white wines (fruity and woody) and 8 types of red wines (fruity and woody) were prepared for this study.

**Table 2:** Concentrations of aroma compounds included in each aroma mixture (fruity and woody).

Aroma Mixture	Aroma Compounds	CAS Number	Concentration in Wine (µg/L)
Woody	Whiskylactone	80041-00-5	165
	Vainillin	121-33-5	55
	Eugenol	97-53-0	8
	Guaiacol	90-05-1	8
	Furaneol	3658-77-3	55
Fruity	2,3-butanedione	431-03-08	1400
	Isoamyl acetate	123-92-2	550
	Ethyl acetate	141-78-6	5000
	Ethyl cinnamate	103-36-6	12
	B-damascenone	23726-93-4	0.3

**Table 3:** ANOVA and Tukey test results showing the effect of PROP taster status on astringency perception considering the different TI parameters in the red and white wines aromatised with woody and fruity aromas.

Wine Type	PROP Phenotype	Astringency in Red Wines				Astringency in White Wines			
		I max	T max	T ext	AUC	I max	T max	T ext	AUC
Woody aromatised wine	T	14.1 a	4.4 a	**104.5 a**	**34,560 a**	11.8 a	5.0 a	91.4 a	22,299 a
	NT	13.9 a	1.8 a	**94.3 b**	**28,326 b**	13.8 a	4.2 a	81.6 a	19,765 a
Fruity aromatised wine	T	13.7 a	**2.6 a**	**103.4 a**	**33,856 a**	**10.9 b**	**4.0 a**	90.9 a	20,397 a
	NT	13.7 a	**1.9 b**	**93.5 b**	**26,776 b**	**13.1 a**	**2.4 b**	80.6 a	21,222 a

T, taster; NT, non-taster. Different letters in bold (a–b) show significant differences among PROP taster status groups from Tukey test (*p* < 0.05).

**Table 4:** ANOVA and Tukey test results showing the effect of PROP taster status on retronasal aroma perception considering the different TI parameters in the red and white wines flavoured with woody and fruity aromas.

Sensory Stimuli	PROP Phenotype	Time–Intensity Parameters in Red Wines				Time–Intensity Parameters in White Wines			
		I max	T max	T ext	AUC	I max	T max	T ext	AUC
Woody aroma	T	14.0 a	4.6 a	100.2 a	28,396 a	12.7 a	3.1 a	97.3 a	24,039 a
	NT	13.7 a	3.5 a	98.3 a	28,310 a	12.4 a	4.7 a	97.2 a	24,544 a
Fruity aroma	T	12.7 a	2.4 a	99.9 a	18,863 a	12.9 a	2.5 a	98.4 a	24,905 a
	NT	13.1 a	2.3 a	99.5 a	26,328 a	13.4 a	2.0 a	96.6 a	29,358 a

T, taster; NT, non-taster. The letter “a” indicates no significant differences among PROP taster status groups from Tukey test (*p* > 0.05).

**Table 5:** ANOVA and Tukey test results showing the effect of the oenological additives on the TI parameters from the astringency evaluation in red and white wines also considering the PROP taster status.

	Astringency									
Wine	PROP Phenotype	Wine Type	Fruity Wine				Woody Wine			
			I max	T max	T ext	AUC	I max	T max	T ext	AUC
	T	CRW	13.3 a	**4.8 a**	100.9 a	28,796 a	15.4 a	9.2 a	105.6 a	35,749 a
Red		GTRW	13.6 a	**2.1 b**	99.0 a	31,371 a	13.4 a	3.4 a	104.1 a	33,543 a
		MRW	13.9 a	**1.9 b**	107.0 a	36,046 a	13.8 a	1.6 a	104.3 a	35,410 a
		ERW	13.9 a	**1.9 b**	106.3 a	38,261 a	13.8 a	2.9 a	104.1 a	33,643 a
	NT	CRW	14.0 a	**2.6 a**	91.7 a	24,492 a	13.9 a	1.8 a	90.6 a	28,702 a
		GTRW	14.0 a	**1.7 b**	101.3 a	31,903 a	14.0 a	2.1 a	95.9 a	25,510 a
		MRW	12.9 a	**1.6 b**	88.7 a	21,175 a	13.8 a	1.8 a	96.6 a	30,489 a
		ERW	13.8 a	**1.8 b**	91.6 a	28,957 a	14.0 a	1.4 a	93.9 a	28,467 a
	T	CWW	**9.0 b**	4.8 a	82.4 a	16,442 a	12.1 a	5.8 a	89.2 a	20,387 a
White		GTWW	**10.4 ab**	6.5 a	83.7 a	18,909 a	13.7 a	2.2 a	96.2 a	25,089 a
		MWW	**12.4 a**	2.7 a	101.1 a	23,279 a	11.2 a	5.8 a	86.3 a	19,368 a
		EWW	**11.7 ab**	2.3 a	95.3 a	22,635 a	10.2 a	6.9 a	92.9 a	23,854 a
	NT	CWW	12.0 a	3.5 a	72.0 a	17,265 a	12.8 a	4.9 a	81.0 a	18,607 a
		GTWW	11.7 a	2.4 a	77.7 a	21,779 a	16.6 a	6.8 a	93.6 a	22,720 a
		MWW	13.3 a	1.9 a	88.6 a	22,720 a	12.7 a	2.3 a	65.0 a	14,221 a
		EWW	15.2 a	1.9 a	83.2 a	22,955 a	12.7 a	2.5 a	82.8 a	22,904 a

T, taster; NT, non-taster. Different letters in bold (a–b) show significant differences from Tukey test (*p* < 0.05) among wine types for each of the PROP taster status groups. Control wine (CW); gallotannin wine (GTW); mannoprotein wine (MW); ellagitannin wine (EW).

**Table 6:** ANOVA and Tukey test results showing the effect of oenological additives on retronasal aroma on the TI parameters from the retronasal aroma evaluation of the red and white wines aromatised with fruity and woody aroma mixtures.

Retronasal Aroma									
Wine	Wine Type	Fruity Aroma				Woody Aroma			
		I max	T max	T ext	AUC	I max	T max	T ext	AUC
	CRW	13.1 a	**2.5 a**	100.5a	27,967 a	13.3 a	2.1 a	99.6 a	27,354 a
Red	GTRW	13.4 a	**1.8 b**	101.0 a	28,541 a	13.1 a	2.1 a	100.2 a	28,167 a
	MRW	13.0 a	**1.7 b**	100 a	2772 a	13.6 a	1.6 a	97.8 a	29,526 a
	ERW	12.1 a	**1.7 b**	100 a	26,411 a	13.4 a	1.7 a	100.7 a	28,336 a
	CWW	13.1 a	**2.9 a**	90.5 a	25,537 a	12.9 a	2.9 a	102.3 a	**25,203 ab**
White	GTWW	13.2 a	**2.5 ab**	98.6a	28,214 a	13.4 a	2.2 a	103.8 a	**29,886 a**
	MWW	13.2 a	**1.5 c**	101.2 a	26,889 a	11.93 a	2.9 a	9.0 a	**20,467 b**
	EWW	13.8 a	**1.8 bc**	99.3 a	27,939 a	12.0 a	3.1 a	91.9 a	**20,999 ab**

Different letters in bold (a–b) show significant differences among wine types from Tukey test (*p* < 0.05). Control wine (CW); gallotannin wine (GTW); mannoprotein wine (MW); ellagitannin wine (EW).

## Data Availability

The data presented in this study are available on request from the corresponding author.

## Supplementary Materials

The following supporting information can be downloaded at: https://www.mdpi.com/article/10.3390/foods12152835/s1, Table S1: Chemical compositions of the control wines without oenological additives (average values); Table S2: Chemical compositions of the oenological additives used in this study.
