# Supplementary material for: Evaluation of the Long-Lasting Flavour Perception after the Consumption of Wines Treated with Different Types of Oenological Additives Considering Individual 6-n-Propylthiouracil Taster Status
Source: Foods. 2023 Jul 26;12(15):2835. doi: 10.3390/foods12152835 (PMC10416954; doi:10.3390/foods12152835)
Supplement: Supplementary file 1 [file foods-12-02835-s001.zip › foods-2500871-supplementary.pdf]

foods MDPI

Supplementary Materials

Evaluation of the Long-Lasting Flavour Perception after the Consumption of Wines Treated with Different Types of Oenological Additives Considering Individual 6-n-Propylthiouracil Taster Status

Rafael I. Velázquez-Martínez [1], Celia Criado [1], Carolina Muñoz-González [1], Julia Crespo [2] and María Ángeles Pozo-Bayón [1,*]

[1] Instituto de Investigacion en Ciencias de la Alimentacion, CSIC-UAM, Nicolas Cabrera 9, 28049 Madrid, Spain; rafael.v.m@csic.es (R.I.V.-M.); celia.criado@csic.es (C.C.); c.munoz@csic.es (C.M.-G.)
[2] Departamento de Investigación Agroambiental, Instituto Madrileño de Investigación y Desarrollo Rural, Agrario y Alimentario (IMIDRA), El Encín, A-2km 38.2, 28805 Alcalá de Henares, Spain; julia.crespo.garcia@madrid.org
* Correspondence: m.delpozo@csic.es; Tel.: +34-910-017-961

Table S1. Chemical compositions of the control wines without oenological additives (average values).

Wine	Grape Varieties	Tartaric Acid (g/L)	Malic Acid (g/L)	Lactic Acid (g/L)	Glucose/ Fructose (g/L)	Ca (mg/L)	K (mg/L)	SO_2L (g/L/)	SO_2T (g/L)	Ethanol (%*V*/*V*)	Titratable Acidity (g/L)	Volatile acid (g/L/	pH
**Red**	**Tempranillo**	2.85	0.03	0,62	0.15	58.23	945.5	56	68	13.9	5.07	0.21	3.56
**White**	**Malvar**	3.47	0.53		0.37	74	378.5	28	72	13.8	5.48	0.45	3.10

Table S2. Chemical compositions of the oenological additives used in this study.

Oenological Additive	Polysaccharide (% Mass)	Protein (% mass)	Total Polyphenols (GAE mg/L)
Gallotannin	--	--	1.4
Ellagitannin	--	--	1.36
Mannoprotein	65	3.05	--

Polysaccharides were determined by using the phenol sulfuric method. Protein determination was carried out using the Kjeldahl method. The concentration of total polyphenols was calculated using the Folin–Ciocalteu method (in mg/L gallic acid equivalents).
